# 4-[(4-Amino­phen­yl)sulfan­yl]aniline

**DOI:** 10.1107/S2414314625007953

**Published:** 2025-09-16

**Authors:** Rao M. Uppu, Ogad A. Agu, Patrick F. Mensah, Guoqiang Li, Frank R. Fronczek

**Affiliations:** a Department of Environmental Toxicology Southern University and A&M College Baton Rouge Louisiana 70813 USA; b Department of Mechanical Engineering Southern University and A&M College Baton Rouge Louisiana 70813 USA; c Department of Mechanical and Industrial Engineering Louisiana State University,Baton Rouge Louisiana 70803 USA; d Department of Chemistry Louisiana State University,Baton Rouge Louisiana 70803 USA; University of Aberdeen, United Kingdom

**Keywords:** crystal structure, 4,4′-bridged dianilines, 4,4′-thio­dianiline, shape-memory polymers and vitrimers

## Abstract

The redetermined structure of the title compound, C_12_H_12_N_2_S, was refined from low-temperature (100 K) single-crystal X-ray diffraction data. Although achiral, the compound crystallizes in Sohncke space group *P*2_1_2_1_2_1_ in a chiral conformation distorted from idealized *C*_2_ symmetry and the dihedral angle between the phenyl groups is 72.01 (7)°. In the extended structure, the NH_2_ substituents participate in N—H⋯S, N—H⋯N, and N—H⋯π inter­actions, leading to a three-dimensional hydrogen-bonded array. These results highlight the role of sulfur bridges in tuning packing inter­actions relevant to polymer design.

## Abstract 2

The redetermined structure of the title compound, C_12_H_12_N_2_S, was refined from low-temperature (100 K) single-crystal X-ray diffraction data. Although achiral, the compound crystallizes in Sohncke space group *P*2_1_2_1_2_1_ in a chiral conformation distorted from idealized *C*_2_ symmetry. In the extended structure, the NH_2_ substituents participate in N—H⋯S, N—H⋯N, and N—H⋯π inter­actions, leading to a three-dimensional hydrogen-bonded array. These results highlight the role of sulfur bridges in tuning packing inter­actions relevant to polymer design.

## Graphical abstract

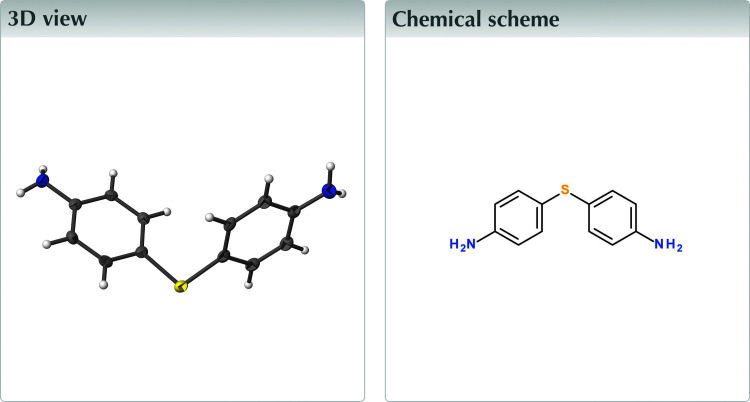

## Structure description

Bridged 4,4′-dianilines are important monomers for high-performance polymers. The nature of the bridging atom or functional group governs rigidity, polarity, and donor strength, which in turn affects curing kinetics, chain packing, and glass transition in dianiline-based polyimides, epoxides and related composites (Ghosh & Mittal, 1996 ▸; Sroog, 1991 ▸). Treated as *para* substituents on each aniline ring, classic Hammett/LFER considerations capture the qu­anti­tative electronic trend, namely –NH– > –O– > –S– > –CH_2_– > –S(=O)– > –SO_2_– (Hansch *et al.*, 1991 ▸). The sulfur bridge in the title compound imparts electronic and steric effects that modulate hydrogen bonding and curing behavior (Mazumder *et al.*, 2023 ▸). Such di­amines are used in polyimides, epoxies, and dynamic polymer networks with shape-memory and vitrimer-like properties (Lendlein & Kelch, 2002 ▸; Xiao *et al.*, 2015 ▸; Winne *et al.*, 2019 ▸; Zi *et al.*, 2021 ▸).

**Figure 1:**
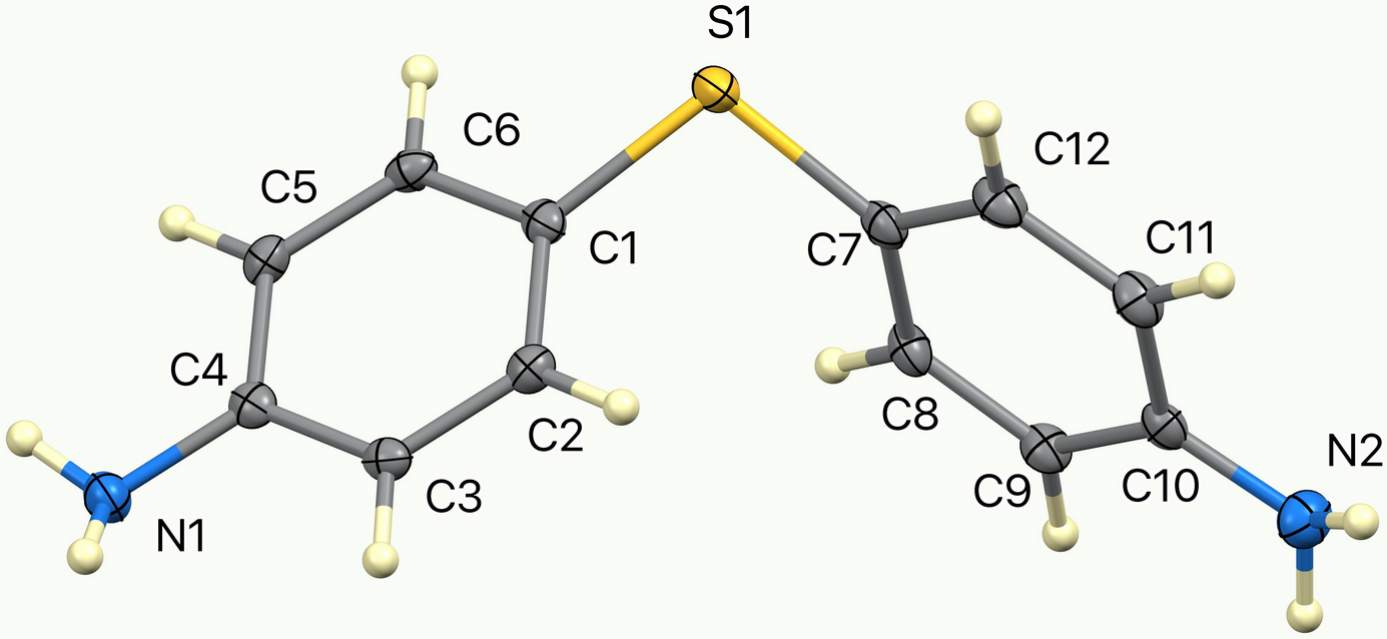
Mol­ecular structure of the title compound with displacement ellipsoids drawn at the 50% probability level.

The crystal structure of the title compound, C_12_H_12_N_2_S, Fig. 1 ▸, was previously reported at room temperature based on film data (Vijayalakshmi & Srinivasan, 1973 ▸; Cambridge Structural Database refcode DAPHSD). The present study refines the structure at 100 K with modern instrumentation, providing more precise mol­ecular geometry and inter­molecular inter­action details.

The mol­ecule crystallizes in the Sohncke space group *P*2_1_2_1_2_1_, adopting a chiral conformation. The phenyl groups are rotated unequally with respect to the central C—S—C plane, giving C_1_ symmetry in the solid state. The dihedral angle between the C1–C6 and C7–C12 phenyl groups is 72.01 (7)°, while the individual dihedral angles with the C1—S1—C7 plane are 34.96 (8) and 52.35 (10)°, respectively. These distortions highlight the conformational flexibility of the sulfanyl bridge. The bond-angle sums at atoms N1 and N2 are 338.3 and 350.2°, respectively, suggesting that the latter atom has more *sp*^2^ electronic character, which may correlate with the fact that the C10—N2 bond [1.377 (3) Å] is shorter than C4—N1 [1.407 (3) Å].

**Figure 2:**
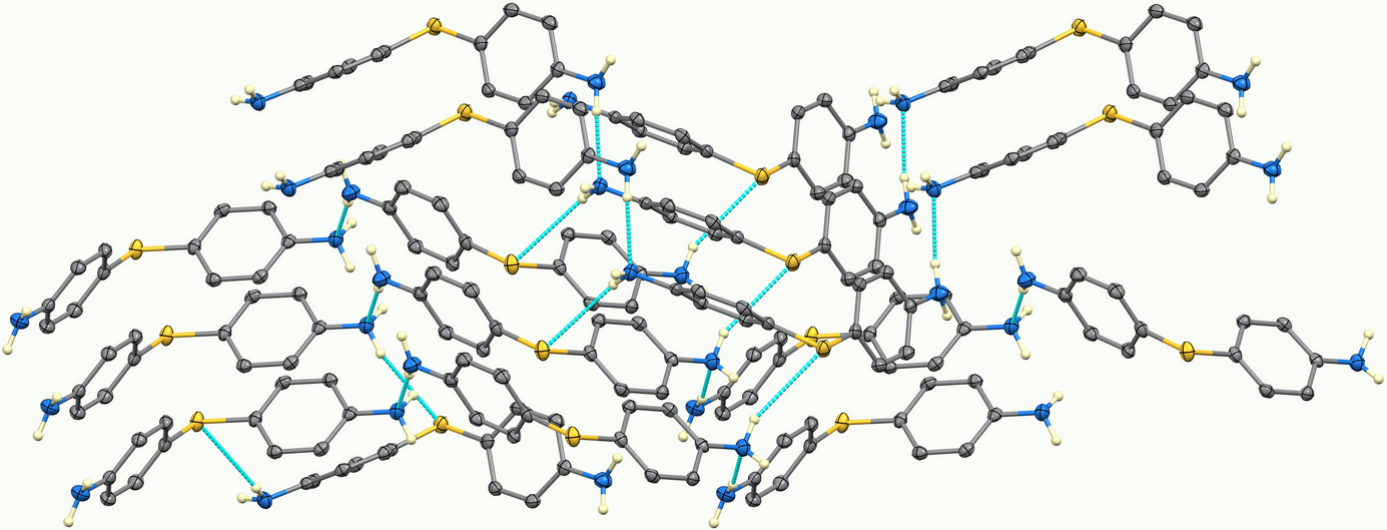
Hydrogen-bonding network in the title compound; only N-bound H atoms are shown.

In the crystal, atom N1 donates a hydrogen bond (Table 1 ▸, Figs. 2 ▸, 3 ▸) to the sulfanyl sulfur atom of an adjacent mol­ecule, while N2 accepts a hydrogen bond from a neighboring NH group. Additionally, one NH hydrogen atom is involved in an N—H⋯π inter­action with a phenyl ring. These inter­actions generate a three-dimensional network of hydrogen bonds and π contacts, contributing to the stability of the packing arrangement. Graph sets for the conventional hydrogen bonds are a 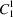
(7) chain in the [100] direction, a 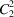
(9) chain in the [010] direction, and a 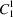
(12) chain in the [001] direction.

The mol­ecular geometry of the title compound reflects the distinctive influence of the sulfanyl bridge. The C—S—C linkage is longer and more flexible than the C—C bond in 4,4′-methyl­enedianiline (Bel’skii *et al.*, 1983 ▸; Gibson *et al.*, 2010 ▸; Uppu *et al.*, 2025*a* ▸) and less bent but electronically softer than the C—O—C bridge in *N*,*N*′-[oxybis(benzene-4,1-di­yl)]diacetamide (Uppu *et al.*, 2025*b* ▸) and 4,4′-oxydianiline (Sharma *et al.*, 2019 ▸; Uppu *et al.*, 2025*c* ▸). In contrast, the SO_2_ bridge in 4,4′-sulfonyl­dianiline (dapsone) (Karle & Karle, 1964 ▸; Uppu & Fronczek, 2025 ▸) introduces polarity and strong hydrogen bonding, markedly altering the packing. Thus, a systematic variation in the bridging unit (NH, O, S, CH_2_, SO, SO_2_) governs bond distances, torsional distortions, and inter­molecular inter­actions, providing a framework for understanding the structure–property relationships of these technologically important dianilines.

## Synthesis and crystallization

4-[(4-Amino­phen­yl)sulfan­yl]aniline (CAS 139–65-1) was purchased from AmBeed (Arlington Heights, IL, USA) with a reported purity of over 97% and used without further purification. Crystals suitable for X-ray analysis were grown by slow cooling hot aqueous solutions, yielding colorless needle-like crystals.

## Refinement

Crystal data, data collection, and structure refinement details are summarized in Table 2 ▸.

## Supplementary Material

Crystal structure: contains datablock(s) I. DOI: 10.1107/S2414314625007953/hb4534sup1.cif

Structure factors: contains datablock(s) I. DOI: 10.1107/S2414314625007953/hb4534Isup2.hkl

Supporting information file. DOI: 10.1107/S2414314625007953/hb4534Isup3.cml

CCDC reference: 2485505

Additional supporting information:  crystallographic information; 3D view; checkCIF report

## Figures and Tables

**Figure 3:**
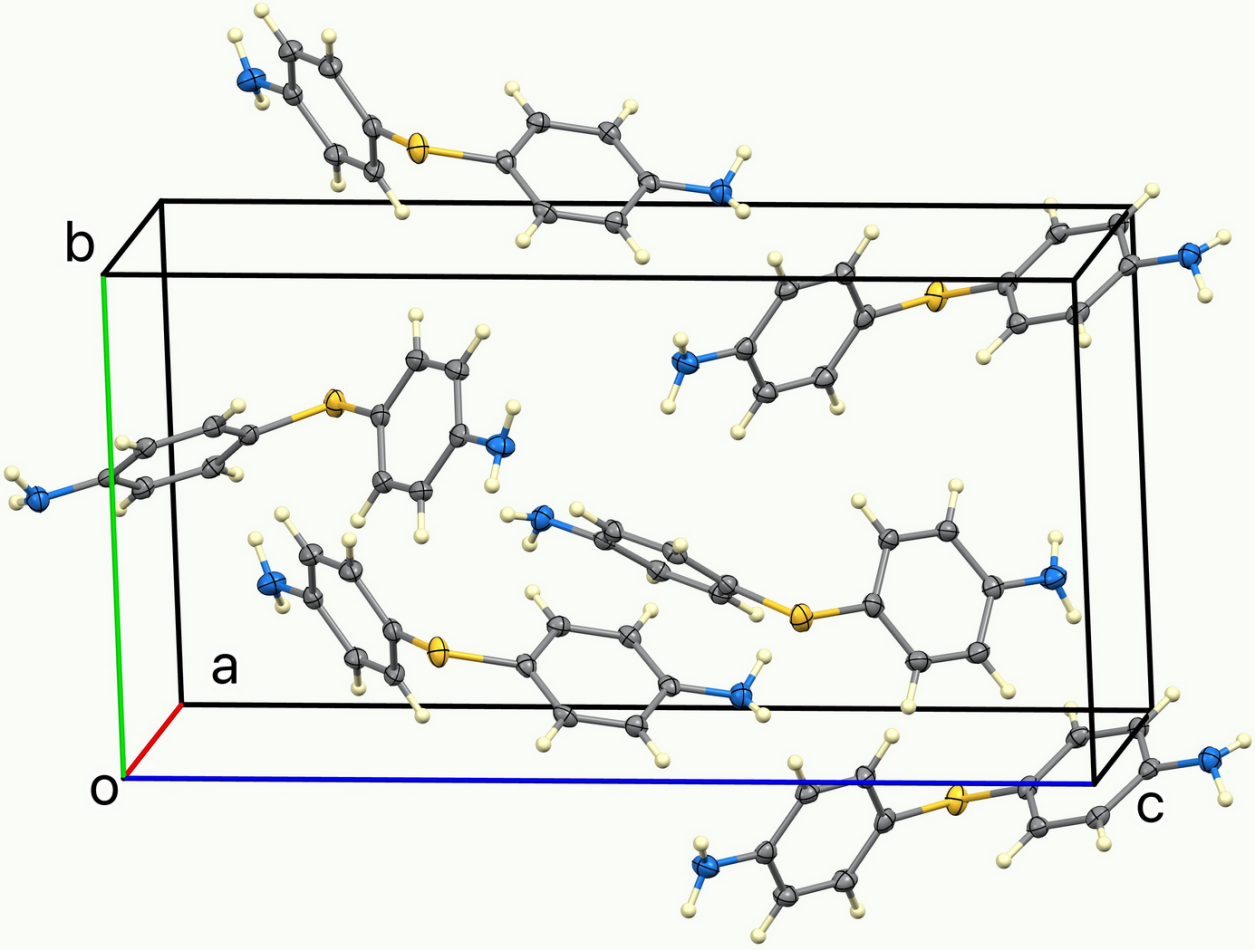
The unit cell of the title compound.

**Table 1:** Hydrogen-bond geometry (Å, °)

*D*—H⋯*A*	*D*—H	H⋯*A*	*D*⋯*A*	*D*—H⋯*A*
N1—H11*N*⋯S1^i^	0.92 (3)	2.88 (3)	3.675 (2)	145 (2)
N1—H12*N*⋯*Cg*2^ii^	0.86 (3)	2.80 (3)	3.553 (2)	147 (3)
N2—H22*N*⋯N1^iii^	0.91 (3)	2.20 (3)	3.095 (3)	167 (3)

Symmetry codes: (i) 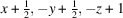; (ii) 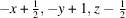; (iii) 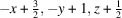.

**Table 2:** Experimental details

Crystal data	
Chemical formula	C_12_H_12_N_2_S
*M*_r_	216.30
Crystal system, space group	Orthorhombic, *P*2_1_2_1_2_1_
Temperature (K)	100
*a*, *b*, *c* (Å)	5.9287 (2), 9.8523 (3), 18.7766 (5)
*V* (Å^3^)	1096.77 (6)
*Z*	4
Radiation type	Cu *K*α
μ (mm^−1^)	2.34
Crystal size (mm)	0.34 × 0.06 × 0.04

Data collection	
Diffractometer	Bruker D8 Venture DUO with Photon III C14
Absorption correction	Multi-scan (*SADABS*; Krause *et al.*, 2015 ▸)
*T*_min_, *T*_max_	0.727, 0.912
No. of measured, independent and observed [*I* > 2σ(*I*)] reflections	13111, 2330, 2215
*R*_int_	0.043
(sin θ/λ)_max_ (Å^−1^)	0.637

Refinement	
*R*[*F*^2^ > 2σ(*F*^2^)], *wR*(*F*^2^), *S*	0.028, 0.070, 1.07
No. of reflections	2330
No. of parameters	148
H-atom treatment	H atoms treated by a mixture of independent and constrained refinement
Δρ_max_, Δρ_min_ (e Å^−3^)	0.15, −0.23
Absolute structure	Flack *x* determined using 858 quotients [(*I*^+^)−(*I*^−^)]/[(*I*^+^)+(*I*^−^)] (Parsons *et al.*, 2013 ▸)
Absolute structure parameter	0.016 (9)

Computer programs: *APEX5* and *SAINT* (Bruker, 2016 ▸), *SHELXT2018/2* (Sheldrick, 2015*a* ▸), *SHELXL2019/1* (Sheldrick, 2015*b* ▸), *Mercury* (Macrae *et al.*, 2020 ▸) and *publCIF* (Westrip, 2010 ▸).

## full crystallographic data

### 4-[(4-Aminophenyl)sulfanyl]aniline Crystal data

**Table:**

C_12_H_12_N_2_S	*D*_x_ = 1.310 Mg m^−^^3^
*M**_r_* = 216.30	Cu *K*α radiation, λ = 1.54184 Å
Orthorhombic, *P*2_1_2_1_2_1_	Cell parameters from 6002 reflections
*a* = 5.9287 (2) Å	θ = 5.1–79.3°
*b* = 9.8523 (3) Å	µ = 2.34 mm^−^^1^
*c* = 18.7766 (5) Å	*T* = 100 K
*V* = 1096.77 (6) Å^3^	Needle, colourless
*Z* = 4	0.34 × 0.06 × 0.04 mm
*F*(000) = 456	

### 4-[(4-Aminophenyl)sulfanyl]aniline Data collection

**Table:**

Bruker D8 Venture DUO with Photon III C14 diffractometer	2215 reflections with *I* > 2σ(*I*)
Radiation source: IµS 3.0 microfocus	*R*_int_ = 0.043
φ and ω scans	θ_max_ = 79.3°, θ_min_ = 4.7°
Absorption correction: multi-scan (SADABS; Krause *et al.*, 2015)	*h* = −7→7
*T*_min_ = 0.727, *T*_max_ = 0.912	*k* = −12→12
13111 measured reflections	*l* = −23→20
2330 independent reflections	

### 4-[(4-Aminophenyl)sulfanyl]aniline Refinement

**Table:**

Refinement on *F*^2^	Hydrogen site location: mixed
Least-squares matrix: full	H atoms treated by a mixture of independent and constrained refinement
*R*[*F*^2^ > 2σ(*F*^2^)] = 0.028	*w* = 1/[σ^2^(*F*_o_^2^) + (0.0356*P*)^2^ + 0.160*P*] where *P* = (*F*_o_^2^ + 2*F*_c_^2^)/3
*wR*(*F*^2^) = 0.070	(Δ/σ)_max_ = 0.001
*S* = 1.07	Δρ_max_ = 0.15 e Å^−^^3^
2330 reflections	Δρ_min_ = −0.23 e Å^−^^3^
148 parameters	Absolute structure: Flack *x* determined using 858 quotients [(*I*^+^)-(*I*^-^)]/[(*I*^+^)+(*I*^-^)] (Parsons *et al.*, 2013)
0 restraints	Absolute structure parameter: 0.016 (9)
Primary atom site location: dual	

### 4-[(4-Aminophenyl)sulfanyl]aniline Special details

**Table:**

Geometry. All esds (except the esd in the dihedral angle between two l.s. planes) are estimated using the full covariance matrix. The cell esds are taken into account individually in the estimation of esds in distances, angles and torsion angles; correlations between esds in cell parameters are only used when they are defined by crystal symmetry. An approximate (isotropic) treatment of cell esds is used for estimating esds involving l.s. planes.
Refinement. All H atoms were located in difference maps and those on C were thereafter treated as riding in geometrically idealized positions with C—H distances 0.95 Å. Coordinates of the NH hydrogen atoms were refined freely. *U*_iso_(H) values were assigned as 1.2*U*_eq_ for the attached atom (1.5 for NH_2_). The absolute structure (Parsons *et al.*, 2013) was determined using 858 quotients, *x* = 0.016 (9).

### 4-[(4-Aminophenyl)sulfanyl]aniline Fractional atomic coordinates and isotropic or equivalent isotropic displacement parameters (Å^2^)

**Table:**

	*x*	*y*	*z*	*U*_iso_*/*U*_eq_	
S1	0.02434 (8)	0.32288 (5)	0.70350 (3)	0.02444 (15)	
N1	0.4024 (3)	0.46157 (19)	0.41708 (10)	0.0235 (4)	
H11N	0.495 (5)	0.397 (3)	0.3971 (14)	0.035*	
H21N	0.801 (5)	0.215 (3)	0.9358 (14)	0.035*	
N2	0.7632 (3)	0.29720 (19)	0.91853 (11)	0.0264 (4)	
H12N	0.296 (6)	0.484 (3)	0.3881 (16)	0.040*	
H22N	0.878 (5)	0.359 (3)	0.9154 (15)	0.040*	
C1	0.1541 (4)	0.3678 (2)	0.62144 (11)	0.0187 (4)	
C2	0.3648 (3)	0.3204 (2)	0.59946 (11)	0.0190 (4)	
H2	0.452849	0.265918	0.630629	0.023*	
C3	0.4461 (3)	0.35248 (19)	0.53226 (11)	0.0185 (4)	
H3	0.590611	0.320796	0.518102	0.022*	
C4	0.3188 (4)	0.43047 (19)	0.48526 (11)	0.0182 (4)	
C5	0.1109 (4)	0.4804 (2)	0.50778 (12)	0.0192 (4)	
H5	0.023750	0.535640	0.476755	0.023*	
C6	0.0302 (4)	0.44981 (19)	0.57538 (11)	0.0193 (4)	
H6	−0.111145	0.485218	0.590355	0.023*	
C7	0.2534 (4)	0.3146 (2)	0.76396 (11)	0.0208 (4)	
C8	0.4022 (4)	0.4232 (2)	0.77322 (11)	0.0210 (5)	
H8	0.385394	0.502546	0.744986	0.025*	
C9	0.5729 (4)	0.41644 (19)	0.82279 (11)	0.0212 (5)	
H9	0.673852	0.490715	0.827738	0.025*	
C10	0.6002 (4)	0.3010 (2)	0.86635 (11)	0.0200 (4)	
C11	0.4482 (4)	0.1934 (2)	0.85710 (11)	0.0236 (4)	
H11	0.461301	0.114997	0.886228	0.028*	
C12	0.2797 (4)	0.1995 (2)	0.80637 (11)	0.0234 (5)	
H12	0.180799	0.124541	0.800321	0.028*	

### 4-[(4-Aminophenyl)sulfanyl]aniline Atomic displacement parameters (Å^2^)

**Table:**

	*U*^11^	*U*^22^	*U*^33^	*U*^12^	*U*^13^	*U*^23^
S1	0.0210 (2)	0.0331 (3)	0.0192 (3)	−0.0047 (2)	0.0034 (2)	−0.0010 (2)
N1	0.0250 (10)	0.0259 (9)	0.0197 (10)	0.0004 (8)	0.0027 (8)	0.0011 (7)
N2	0.0280 (10)	0.0207 (9)	0.0303 (11)	0.0011 (8)	−0.0029 (9)	0.0037 (8)
C1	0.0200 (10)	0.0188 (9)	0.0172 (10)	−0.0034 (8)	0.0010 (8)	−0.0038 (8)
C2	0.0194 (9)	0.0168 (9)	0.0209 (11)	0.0007 (8)	−0.0022 (8)	−0.0014 (8)
C3	0.0162 (9)	0.0185 (9)	0.0207 (10)	−0.0008 (7)	0.0012 (8)	−0.0045 (7)
C4	0.0206 (10)	0.0150 (9)	0.0191 (11)	−0.0029 (7)	0.0008 (9)	−0.0040 (8)
C5	0.0198 (10)	0.0183 (9)	0.0195 (11)	0.0017 (7)	−0.0046 (9)	−0.0017 (8)
C6	0.0154 (9)	0.0198 (9)	0.0227 (11)	0.0020 (8)	−0.0012 (9)	−0.0057 (8)
C7	0.0241 (10)	0.0229 (9)	0.0154 (10)	−0.0020 (9)	0.0051 (8)	−0.0029 (8)
C8	0.0284 (11)	0.0184 (9)	0.0163 (11)	−0.0026 (8)	0.0037 (9)	0.0012 (8)
C9	0.0255 (11)	0.0170 (9)	0.0211 (11)	−0.0029 (8)	0.0024 (9)	−0.0012 (8)
C10	0.0219 (10)	0.0204 (9)	0.0176 (10)	0.0018 (8)	0.0048 (8)	−0.0024 (8)
C11	0.0305 (11)	0.0174 (9)	0.0230 (11)	−0.0002 (9)	0.0035 (9)	0.0021 (8)
C12	0.0285 (10)	0.0184 (9)	0.0233 (12)	−0.0045 (8)	0.0051 (9)	−0.0011 (8)

### 4-[(4-Aminophenyl)sulfanyl]aniline Geometric parameters (Å, º)

**Table:**

S1—C7	1.772 (2)	C4—C5	1.393 (3)
S1—C1	1.778 (2)	C5—C6	1.390 (3)
N1—C4	1.407 (3)	C5—H5	0.9500
N1—H11N	0.92 (3)	C6—H6	0.9500
N1—H12N	0.86 (3)	C7—C12	1.394 (3)
N2—C10	1.377 (3)	C7—C8	1.398 (3)
N2—H21N	0.90 (3)	C8—C9	1.376 (3)
N2—H22N	0.91 (3)	C8—H8	0.9500
C1—C6	1.393 (3)	C9—C10	1.410 (3)
C1—C2	1.397 (3)	C9—H9	0.9500
C2—C3	1.387 (3)	C10—C11	1.402 (3)
C2—H2	0.9500	C11—C12	1.382 (3)
C3—C4	1.392 (3)	C11—H11	0.9500
C3—H3	0.9500	C12—H12	0.9500
			
C7—S1—C1	103.60 (10)	C5—C6—C1	120.75 (19)
C4—N1—H11N	115.4 (16)	C5—C6—H6	119.6
C4—N1—H12N	112 (2)	C1—C6—H6	119.6
H11N—N1—H12N	111 (3)	C12—C7—C8	118.7 (2)
C10—N2—H21N	117.1 (18)	C12—C7—S1	119.29 (16)
C10—N2—H22N	117.3 (19)	C8—C7—S1	121.90 (16)
H21N—N2—H22N	116 (3)	C9—C8—C7	120.76 (18)
C6—C1—C2	118.9 (2)	C9—C8—H8	119.6
C6—C1—S1	117.02 (16)	C7—C8—H8	119.6
C2—C1—S1	124.03 (17)	C8—C9—C10	121.04 (19)
C3—C2—C1	120.20 (19)	C8—C9—H9	119.5
C3—C2—H2	119.9	C10—C9—H9	119.5
C1—C2—H2	119.9	N2—C10—C11	121.24 (19)
C2—C3—C4	120.95 (19)	N2—C10—C9	121.04 (19)
C2—C3—H3	119.5	C11—C10—C9	117.6 (2)
C4—C3—H3	119.5	C12—C11—C10	121.15 (19)
C3—C4—C5	118.8 (2)	C12—C11—H11	119.4
C3—C4—N1	120.41 (19)	C10—C11—H11	119.4
C5—C4—N1	120.7 (2)	C11—C12—C7	120.7 (2)
C6—C5—C4	120.4 (2)	C11—C12—H12	119.7
C6—C5—H5	119.8	C7—C12—H12	119.7
C4—C5—H5	119.8		
			
C7—S1—C1—C6	−146.49 (16)	C1—S1—C7—C12	−129.04 (17)
C7—S1—C1—C2	36.99 (19)	C1—S1—C7—C8	54.41 (19)
C6—C1—C2—C3	−1.3 (3)	C12—C7—C8—C9	0.6 (3)
S1—C1—C2—C3	175.15 (15)	S1—C7—C8—C9	177.12 (16)
C1—C2—C3—C4	−0.9 (3)	C7—C8—C9—C10	−1.0 (3)
C2—C3—C4—C5	2.4 (3)	C8—C9—C10—N2	−176.5 (2)
C2—C3—C4—N1	−179.57 (18)	C8—C9—C10—C11	0.3 (3)
C3—C4—C5—C6	−1.6 (3)	N2—C10—C11—C12	177.7 (2)
N1—C4—C5—C6	−179.62 (18)	C9—C10—C11—C12	0.9 (3)
C4—C5—C6—C1	−0.7 (3)	C10—C11—C12—C7	−1.4 (3)
C2—C1—C6—C5	2.1 (3)	C8—C7—C12—C11	0.7 (3)
S1—C1—C6—C5	−174.60 (15)	S1—C7—C12—C11	−175.99 (16)

### 4-[(4-Aminophenyl)sulfanyl]aniline Hydrogen-bond geometry (Å, º)

**Table:**

*D*—H···*A*	*D*—H	H···*A*	*D*···*A*	*D*—H···*A*
N1—H11*N*···S1^i^	0.92 (3)	2.88 (3)	3.675 (2)	145 (2)
N1—H12*N*···*Cg*2^ii^	0.86 (3)	2.80 (3)	3.553 (2)	147 (3)
N2—H22*N*···N1^iii^	0.91 (3)	2.20 (3)	3.095 (3)	167 (3)

Symmetry codes: (i) *x*+1/2, −*y*+1/2, −*z*+1; (ii) −*x*+1/2, −*y*+1, *z*−1/2; (iii) −*x*+3/2, −*y*+1, *z*+1/2.
